# Baculovirus entire ORF1629 is not essential for viral replication

**DOI:** 10.1371/journal.pone.0221594

**Published:** 2019-08-22

**Authors:** Won Seok Gwak, See Nae Lee, Jae Bang Choi, Hyun Soo Kim, Beom Ku Han, Sung Min Bae, Yeon Ho Je, Soo Dong Woo

**Affiliations:** 1 Department of Agricultural Biology, College of Agriculture, Life & Environment Science, Chungbuk National University, Cheongju, Korea; 2 Optipharm Inc., Osong, Korea; 3 Diagnostic Development 2 Team, ImmuneMed Inc., Chuncheon, Korea; 4 Department of Agricultural Biotechnology, College of Agriculture & Life Science, Seoul National University, Seoul, Korea; Fundacao Oswaldo Cruz, BRAZIL

## Abstract

It is generally accepted that ORF1629 is essential for baculovirus replication, which has enabled isolation of recombinant viruses in a baculovirus expression system using linearized viral DNA. ORF1629-defective viruses cannot replicate in insect cells; only recombinant virus with complete ORF1629 restoration by recombination can propagate, allowing for pure isolation and the development of bacmids for easy selection of recombinant viruses. We inadvertently found proliferation in insect cells of a bacmid lacking a complete ORF1629. PCR indicated no other viruses but a lack of complete ORF1629 in the proliferated bacmid, suggesting that the baculovirus propagated without a complete ORF1629. Lack of ORF1629 decreased the virus growth rate and yield; it also increased the occlusion body (OB) size but decreased its yield. These results were confirmed for *Autographa californica* multicapsid nucleopolyhedrovirus (AcMNPV) and *Bombyx mori* NPV (BmNPV). Thus, entire ORF1629 is not essential for viral replication, though it does affect the virus growth rate, yield, and size and OB production.

## Introduction

The baculovirus expression system (BES) is widely used for the production of useful proteins due to high productivity and excellent post-translational modification capability [1–4]. To produce a useful protein using BES, it is necessary to generate a target gene-carrying recombinant virus via homologous recombination between viral DNA and transfer-vector DNA. However, because such gene recombination efficiencies are very low, isolation of recombinant viruses is difficult [4]. The use of linearized viral DNA with a defective ORF1629 solves this problem, which has allowed BES to become popular [5–6]. In this case, the virus can be propagated only when the linearized viral DNA resumes a circular shape and the ORF1629 gene, which is essential for viral replication [1], is completely restored [5–6]. This restoration is possible only through recombination of the viral DNA and the target gene, facilitating isolation of the recombinant virus. Based on this technology, various forms of bacmids have been produced for the generation and isolation of recombinant viruses [1]. However, this technique is not a perfect approach for pure isolation of recombinant viruses. In cases in which a recombinant virus is generated using linearized viral DNA and defective ORF1629, a plaque assay or end-point dilution method, which is another method for separation, is also needed. Thus, due to accidental recovery of the linearized viral DNA, it is possible that a non-target virus will be allowed to grow and that additional separation will be required to obtain the intended recombinant virus.

ORF1629 is expressed by all alpabaculoviruses; it is reported to be a structural protein of occlusion-derived viruses and is generally accepted as essential to viral replication [1, 7–9]. Therefore, viral DNA lacking complete ORF1629 cannot be propagated. Only a virus with complete ORF1629 substitution in the genome through recombination can be propagated, and this approach been thus used for the pure isolation of recombinant virus in BES. In this study, we inadvertently found proliferation of a bacmid deficient in ORF1629 in insect cells. Accordingly, we experimentally analyzed whether entire ORF1629 is indeed essential for viral replication and investigated the effect of defective ORF1629 on virus proliferation.

## Materials and methods

### Viruses and cells

*Spodoptera frugiperda* 9 (Sf9) and *Bombyx mori* 5 (Bm5) cells were maintained at 27°C in SF900 II serum-free medium (Gibco, USA) and TC-100 insect medium (WelGENE, Korea) supplemented with 10% fetal bovine serum. Wild-type *Autographa californica* multicapsid nucleopolyhedrovirus (AcMNPV) C6 and *Bombyx mori* NPV (BmNPV) K1 strains were used in this study (S1 Fig). Recombinant vApEGFP and vBpEGFP expressing the enhanced green fluorescent protein (EGFP) gene under the control of the *polyhedrin* promoter were used as controls (S1 Fig). Routine cell culture maintenance and virus production procedures were performed according to published procedures [4]

### Bacmid and transfection

Bacmids bApGOZA and bBpGOZA, which have ORF1629-defective viral genomes, were used in this study [10–12] (S1 Fig). Bacmid transfection was performed using the Cellfectin II™ (Invitrogen, USA) reagent according to the manufacturer’s instructions.

### PCR and RT-PCR

**Fig 1:**
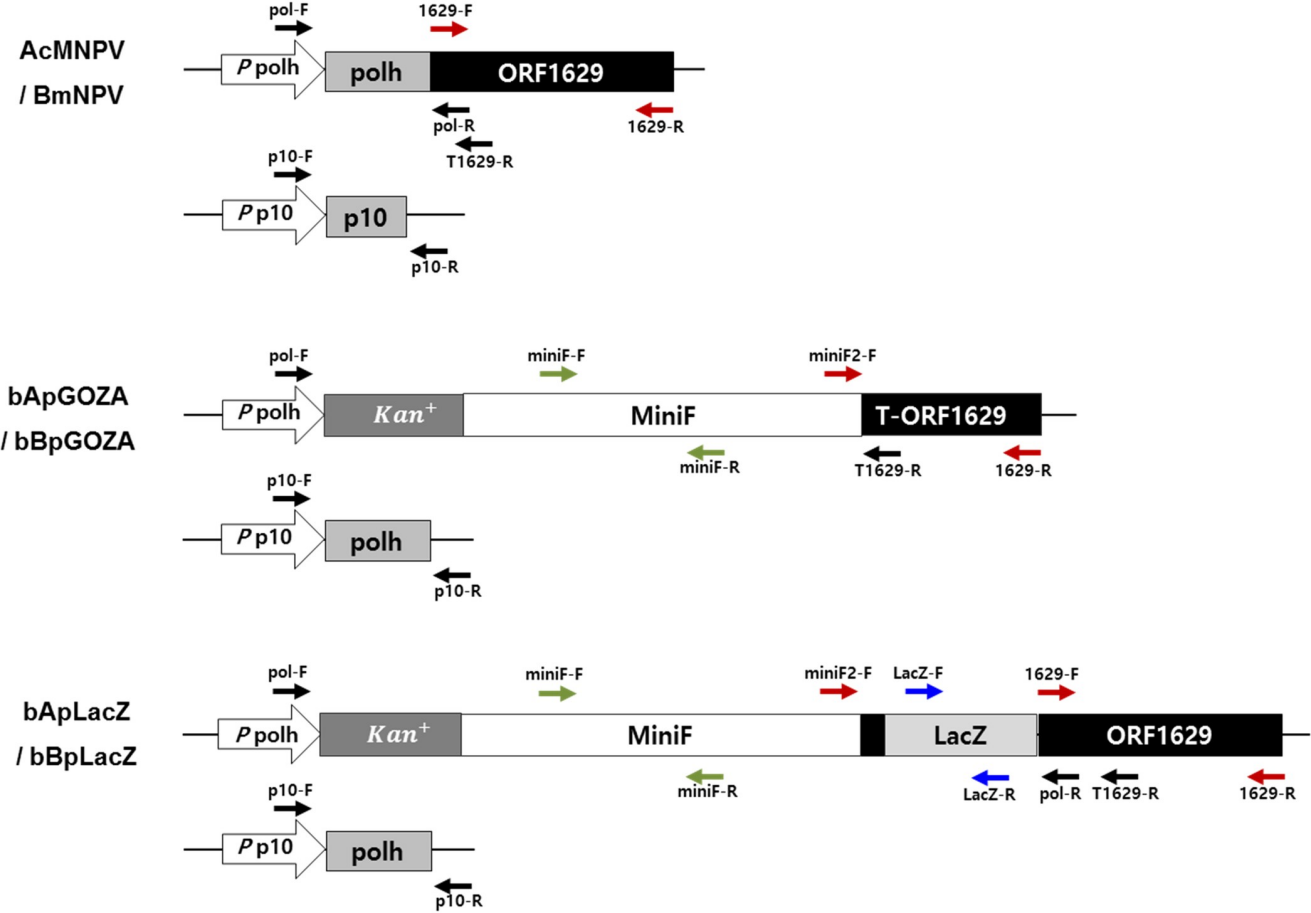
Position of each primer used for PCR amplification.

PCR was performed using the primers shown in Table 1, and the position of each primer is indicated in Fig 1. Primers for polyhedrin, p10 and ORF1629 genes correspond to upstream and downstream regions, whereas primers for LacZ and Mini-F correspond to sites within each coding region. Total RNA was extracted from infected cell cultures with the Viral Gene-spinTM kit (iNtRON Biotechnology, Korea), as recommended by the manufacturer, and used as a template for cDNA synthesis with an RNA LA PCR Kit (TaKaRa, Japan). PCR amplification was performed using AccuPower PCR Premix (Bioneer, Korea). The PCR products were analyzed on a 1% agarose gel and sequenced after cloning into the pMD20-T vector (Takara, Japan).

**Table 1:** Primers used in this study.

Name of Primer	Primer Sequence ^a^
**pol-F**	5′- CAGTTTTGTAATAAAAAAACCTATAAAT -3′
**pol-R**	5′- GTAAATCAACAACGCACAGAATCTAGCGC -3′
**1629-F**	5′- TTAAGCGCTAGATTCTGTGCGT -3′
**1629-R**	5′- GCGTAGCATTTGTGGTGGC -3′
**T1629-R**	5′- CGCCCGATGTTAAATATGTCCAAGC -3′
**LacZ-F**	5′- CTGGCACGACAGGTTTCCCGACTGG -3′
**LacZ-R**	5′- CCATTCGCCATTCAGGCTGCGCAAC -3′
**p10-F**	5′- ACTATGAAATTATGCATTTGAGGATGCC -3′
**p10-R**	5′- ATTATAAAACAATTGATTTGTTATTTTAAAAACG -3′
**miniF-F**	5′- TTATCTAATCTCCCAGCGTGGTT -3′
**miniF-R**	5′- ATGTTCAGAATGAAACTCATGGAA -3′
**miniF2-F**	5′- ATCAGCGCGCAAATACGC -3′

^a^Position of each primer is shown in Fig 1.

### Time course of virus growth

Cells were infected with viruses at a multiplicity of infection (MOI) of 1 plaque-forming unit (PFU) per cell in 6-well culture plates containing 1.0 × 10^6^ cells/well. Following the 1-hr virus adsorption period, the cells were washed, supplied with fresh medium, and incubated at 27°C. At various times after inoculation, the culture supernatant containing budded viruses was removed, centrifuged at 1,000 rpm for 10 min, and stored at -20°C until use. End-point dilution analysis for purification and titration of the virus stock was carried out according to a published procedure [13].

### Occlusion body morphology and yield

A drop of purified occlusion body (OB) solution of each virus was placed on grids and air-dried. The grids were coated with carbon and stained with gold, and the samples were observed under a scanning electron microscope (Ultra Plus, Carl Zeiss, Germany). For quantification of total produced OBs, cells were infected with virus at an MOI of 1 PFU per cell. The virus-infected cells were floated by gentle pipetting and harvested at 3, 4 and 5 days p. i.; 1% sodium dodecyl sulfate was added to the cultures to release OBs and incubated at 37°C for 30 min. The released OBs were quantified using a hemocytometer.

## Results

### Proliferation of a bacmid deficient in ORF1629

**Fig 2:**
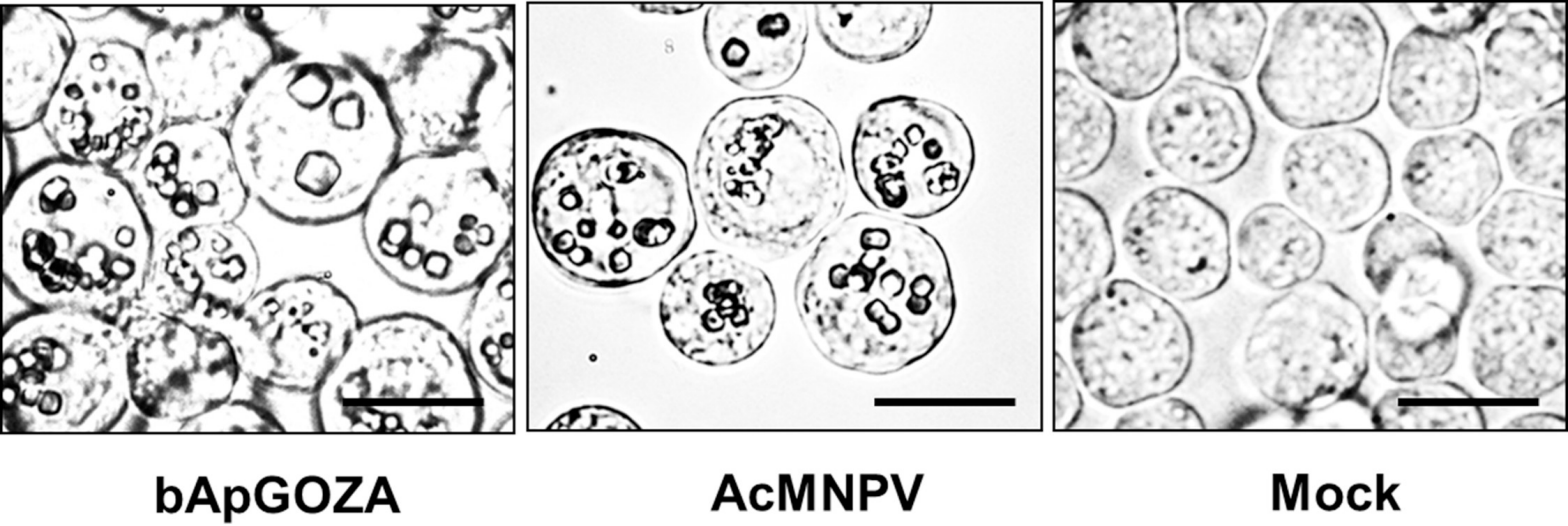
Phase-contrast micrographs of Sf9 cells transfected or infected with bApGOZA or AcMNPV, respectively, at 7 days. Cells were transfected with bApGOZA DNA or infected with AcMNPV at an MOI of 1 PFU/cell. Bar markers represent 20 ㎛.

The proliferation of bApGOZA, an AcMNPV bacmid lacking complete ORF1629, in insect cells was found accidentally (Fig 2). This finding occurred at 7 days after transfection of bApGOZA into cells. The same results were shown in experiments repeated three times or more. As entire ORF1629 is known to be essential for viral replication, proliferation of a bacmid lacking the gene was considered to be impossible. Nonetheless, proliferation of the virus was confirmed and named vApGOZA, and further analysis was performed.

### Identification of viral contamination

**Fig 3:**
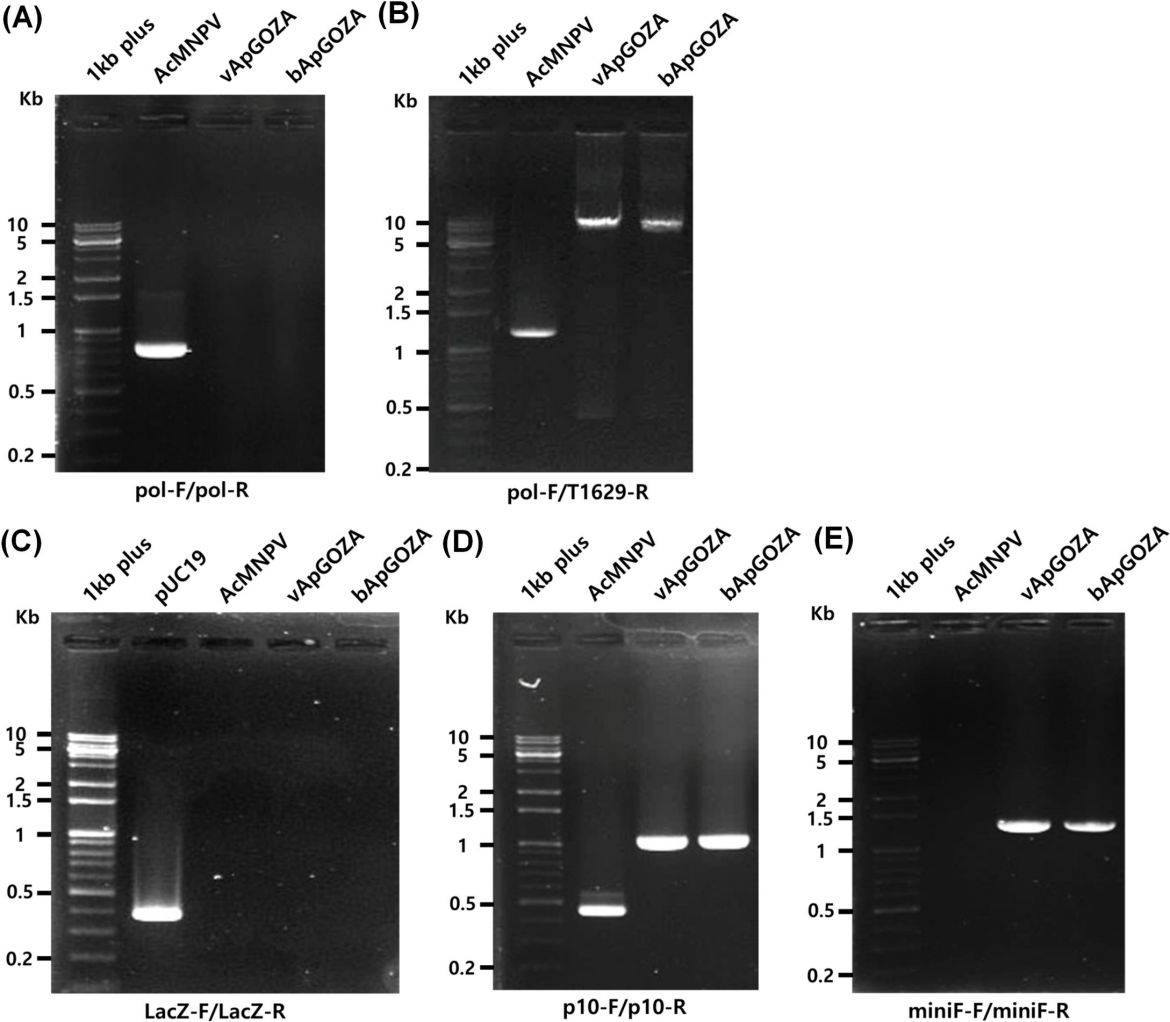
PCR analysis for the identification of viral contamination. PCR amplifications were performed for AcMNPV, vApGOZA, bApGOZA and pUC19 DNA using each primer shown in Fig 1 and Table 1.

It was first ascertained whether the virus proliferated by bApGOZA was due to contamination of other viruses. To check for contamination of wild-type AcMNPV, PCR was performed for the polyhedrin gene region. A PCR product corresponding to a polyhedrin gene of approximately 0.8 kb in size was confirmed for AcMNPV, but PCR products for bApGOZA and vApGOZA were not obtained (Fig 3A). PCR was reperformed using a reverse primer in the truncated ORF1629 region, with a product of approximately 1.2 kb for AcMNPV and approximately 8 kb for both bApGOZA and vApGOZA (Fig 3B). These results indicate that the growth of bApGOZA was not due to AcMNPV contamination. To exclude contamination of bApLacZ during the generation of bApGOZA [12], lacZ gene-specific PCR was performed; a PCR product of approximately 0.4 kb was observed only for pUC19 used as a control (Fig 3C). Thus, the growth of bApGOZA was not due to contamination by bApLacZ. In addition, PCR analysis of the p10 region was performed, confirming the expected products of approximately 1.0 kb for the polyhedrin gene of both bApGOZA and vApGOZA and approximately 0.45 kb for the p10 gene of AcMNPV (Fig 3D). The mini-F region was identified only in bApGOZA and vApGOZA (Fig 3E). These results confirm that vApGOZA has the same structure as bApGOZA for both polyhedrin and p10 gene loci.

### Confirmation of the lack of ORF1629

**Fig 4:**
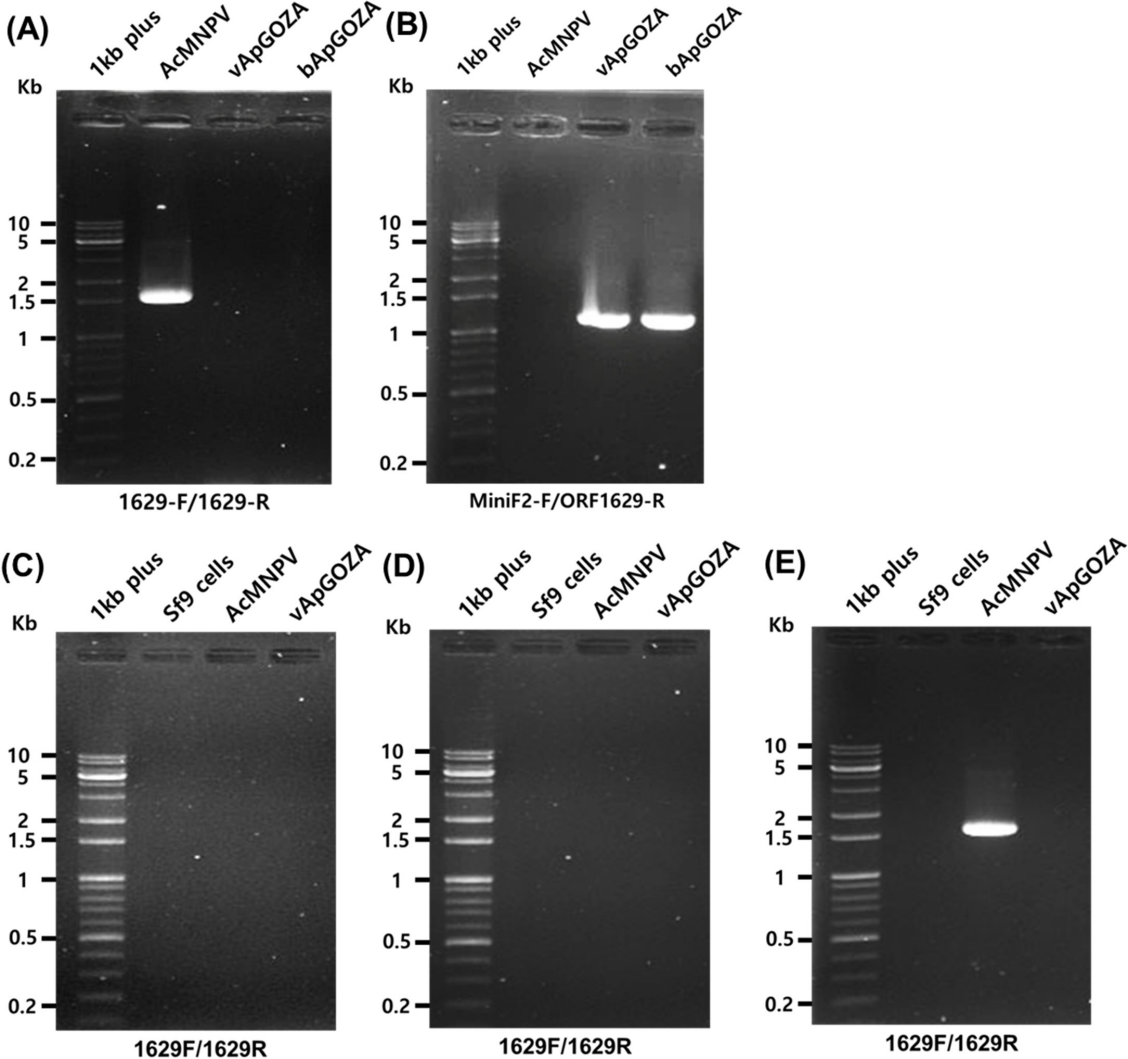
PCR and RT-PCR analyses for ORF1629. **PCR amplifications were performed for AcMNPV, vApGOZA and bApGOZA DNA using each primer shown Fig 1 and Table 1 (A, B).** Sf9 cells were infected with AcMNPV or vApGOZA at an MOI of 5 PFU/cell. Total RNA from infected cells was collected and subjected to RT-PCR, and the products were analyzed by electrophoresis on 1% agarose gels. PCR amplification was performed on DNase-treated total RNA (C). RT-PCR was performed on the cDNA synthesized using truncated (D) and complete (E) ORF1629 gene-specific primers.

As it was verified that the proliferation of bApGOZA was not due to contamination of other viruses and that vApGOZA had a similar genome structure to bApGOZA, further analysis was performed for ORF1629. A complete ORF1629 was found to exist only in AcMNPV (Fig 4A), whereas an incomplete ORF1629 was identified in bApGOZA and vApGOZA (Fig 4B). RT-PCR for ORF1629 was then conducted to confirm the possibility that ORF1629 exists at and is expressed from other loci in vApGOZA: expression of ORF1629 was found only for AcMNPV and not vApGOZA (Fig 4C–4E). These results show that vApGOZA has the same incomplete ORF1629 as bApGOZA. Because virus proliferation was possible with bApGOZA, it was determined whether proliferation occurred for bBpGOZA, a BmNPV bacmid, produced by the same method. The formation of OB at approximately 7 days after transfection of bBpGOZA in insect cells was found (S2 Fig). The virus generated by bBpGOZA was named vBpGOZA, and its lack of ORF1629 was confirmed (S3 Fig). These results suggest that entire ORF1629 is not essential for viral replication via AcMNPV and BmNPV in insect cells.

### Influence of ORF1629 on virus growth

**Fig 5:**
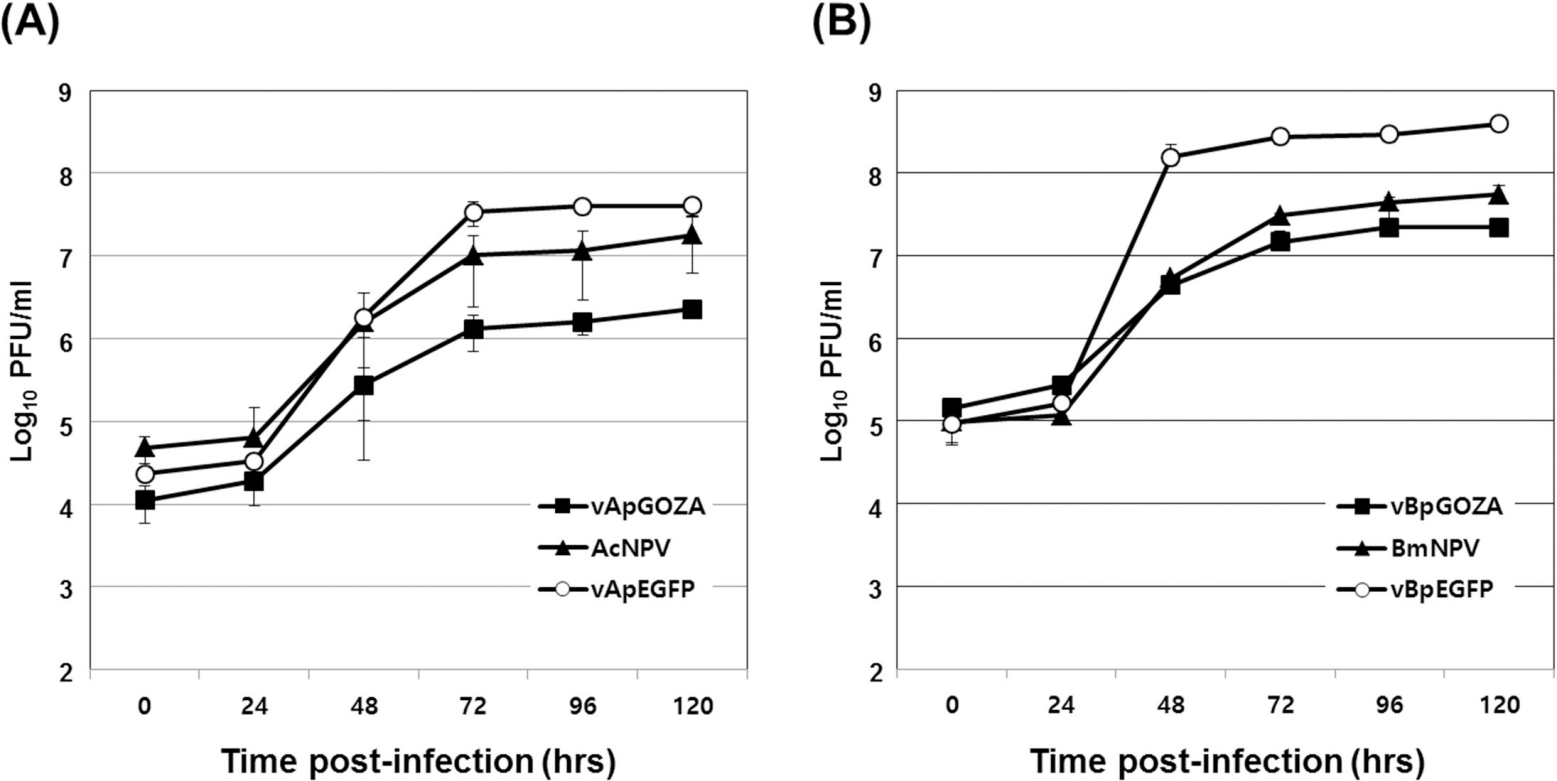
Comparison of viral growth between wild-type and recombinant viruses. Sf9 (A) and Bm5 (B) cells were infected with each virus at an MOI of 1 PFU/cell. Cell culture supernatants were harvested and titrated using TCID_50_ end-point dilution assays. The bars indicate the mean ± SE (n = 3).

Because entire ORF1629 was not essential for virus replication from our results, its influence on virus proliferation was also investigated. We first examined the effects of defective ORF1629 on virus growth and yield and found that rate of growth of both vApGOZA and vBpGOZA was slowed compared with that of the wild-type virus (Fig 5). In addition, virus yield was approximately 8 times lower for vApGOZA and 2.5 times lower for vBpGOZA than for wild-type at 5 days post-infection. These results suggest that ORF1629 affects the formation of budded virus (BV) such that the growth rate and yield are lower; moreover, the effect is stronger with AcMNPV than BmNPV.

### Influence of ORF1629 on occlusion body formation

**Fig 6:**
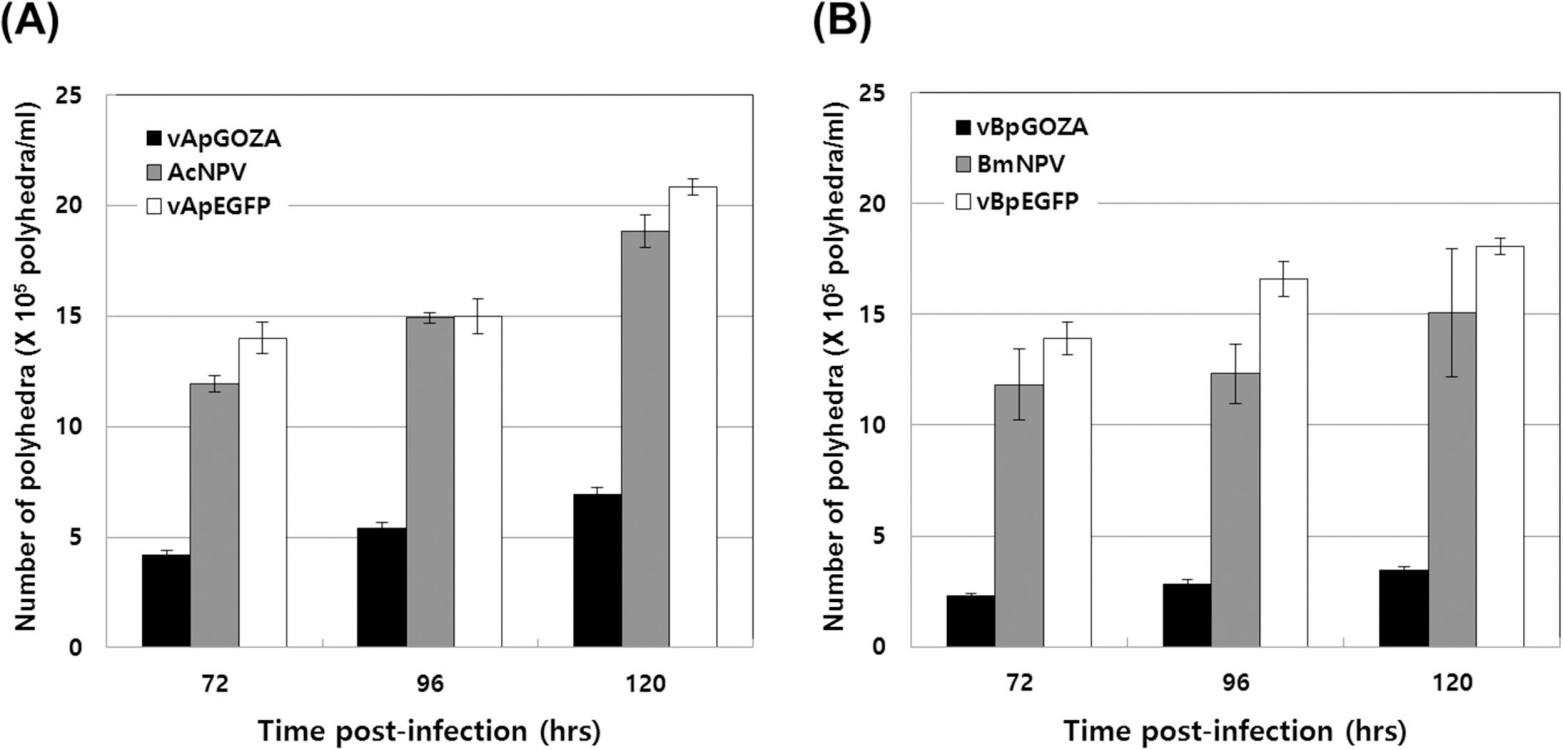
Comparison of OB yields for each virus. Sf9 (A) and Bm5 (B) cells infected with each virus at an MOI of 1 PFU/cell. Yield of total produced OBs was determined using a hemocytometer at 3, 4 and 5 days p. i.

Because ORF1629 is known to be important for BV and occlusion-derived virus (ODV) formation (Pham et al., 1993; Russell et al., 1997; Vialard and Richardson, 1993), the influence of defective ORF1629 on OB formation was investigated. The size of OBs was observed in vApGOZA- and vBpGOZA-infected cells, and their shapes and sizes were compared with those of wild-type viruses (S4 Fig). No significant difference in shape from wild-type was found for either virus, though they did exhibit a large difference in size. The OB size of vApGOZA was larger, approximately 2.3–3.3 μm, than the 1.5–2.2 μm of AcMNPV and 1.4–2.0 μm of vApEGFP used as control viruses. In the case of vBpGOZA, the OB size was larger, at 1.9–4.3 μm, compared to 1.5–3.3 μm for BmNPV and 1.9–2.8 μm for vBpEGFP. Although these results may be due to reduced viral growth or ODVs related to ORF1629, the reason remains unclear. Regarding the yield of OBs, vApGOZA and vBpGOZA produced lower yields, 2.7- to 3.4- and 4.4- to 6.1-fold lower, than did control viruses (Fig 6).

## Discussion

Previous reports have shown that entire ORF1629 is essential for viral replication, and therefore deficient viruses are unable to propagate [6]. This feature has been useful for selecting recombinant viruses. NPV genes are classified as essential and nonessential depending on whether the virus is capable of proliferating in cultured cells without that gene [14–15]. When a gene is deleted but the virus is able to grow, it is considered a nonessential gene; however, these classification criteria are based on experimental results, and they were not been assessed in relation to the original function of the gene. ORF1629 is reported to be a structural protein of both BVs and ODVs, yet the importance of its function remains unknown [7–9]. Regardless, entire ORF1629 is considered to be an essential gene because propagation of an ORF1629-deficient virus does not occur. Because the gene involved in viral particle formation is expected to have a large negative influence on the proliferation of viruses, it has been presumed that researchers could not confirm propagation of a virus lacking the ORF1629 gene. In particular, even if a recombinant virus lacking the complete ORF1629 gene could be produced, the process would have been very inefficient, and the proliferation efficiency would be expected to be low due to negative effects on proliferation. As a result, it can be inferred that even if viral proliferation did occur, it would not be confirmed. In the present study, a large amount of viral DNA (3ug) lacking 407 nucleotides (3' terminus 1223 to 1629) of the ORF1629 gene in the bacmid form (S5 Fig) was transfected in great quantities, thereby overcoming the problem of low recombination efficiency of viral DNA in general recombination process, and it was thus possible to generate a relatively large amount of virus.

In our study, the defective ORF1629 was confirmed by PCR and RT-PCR, though this was indirectly observed by BV production and OB formation. The lack of complete ORF1629 for the formation of BVs markedly reduced the BV growth rate to approximately 8 times lower than that of the wild-type virus. In addition, the increase in size and the decrease in yield of OBs suggest that ORF1629 does participate in ODV formation and also influences the interaction between ODVs and polyhedrin. As FP25 is reported to be mainly involved in the production of fewer OBs [16–17], mutation in this gene was assessed, but no mutation was observed.

In conclusion, entire ORF1629 is not essential for viral growth, though it does affect the virus growth rate, yield, and size as well as production of OBs. These effects may be the result of the influence that ORF1629 has on the formation of both BVs and ODVs. Although we could not determine whether or not the entire ORF1629 is unnecessary for the viral replication, it would be verified through further study.

## Supporting information

S1 Fig Schematic diagram of bacmids and viruses used in our study. (TIF)

S2 Fig Phase-contrast micrographs of Bm5 cells transfected or infected with bBpGOZA or BmNPV, respectively, at 7 days. Cells were transfected with bBpGOZA DNA or infected with BmNPV at an MOI of 1 PFU/cell. Bar markers represent 20 ㎛.
(TIF)

S3 Fig PCR analysis for ORF1629. PCR amplifications were performed for BmNPV, vBpGOZA and bBpGOZA DNA using each primer shown Fig 1 and Table 1.
(TIF)

S4 Fig **Scanning electron micrographs of purified OBs of AcMNPV (A), BmNPV (B), vApEGFP (C), vBpEGFP (D), vApGOZA (E) and vBpGOZA (F).** Bar markers represent 1 *μ*m.
(TIF)

S5 Fig Nucleotide sequences surrounding polyhedrin promoter regions of bApGOZA. **ORF603, miniF, kanamycin resistance and defective ORF1629 genes were shown.** Nucleotides of 1223 to 1629 from ORF1629 were missed.
(DOCX)
