# Supplementary figures and images for: Baculovirus entire ORF1629 is not essential for viral replication
Source: PLoS One. 2019 Aug 22;14(8):e0221594. doi: 10.1371/journal.pone.0221594 (PMC6706055; doi:10.1371/journal.pone.0221594)

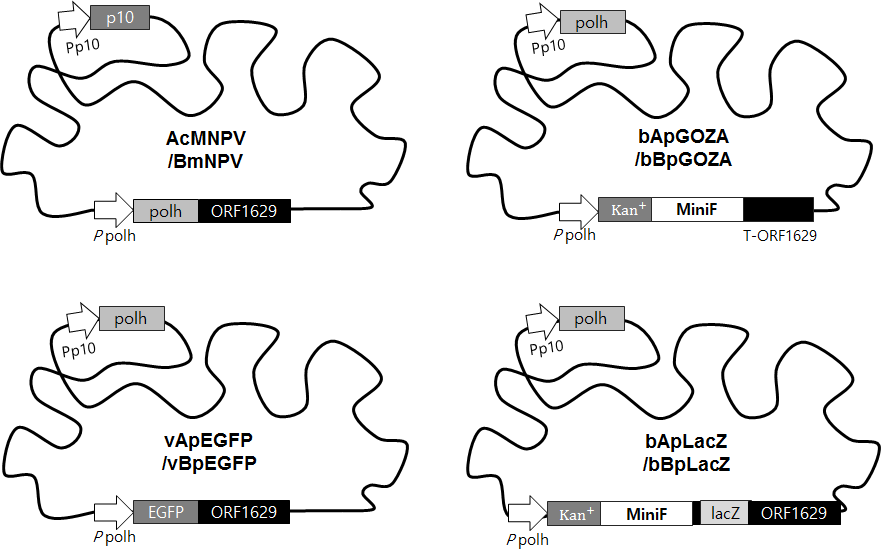

Supplement: S1 Fig — (TIF) [file pone.0221594.s001.tif]

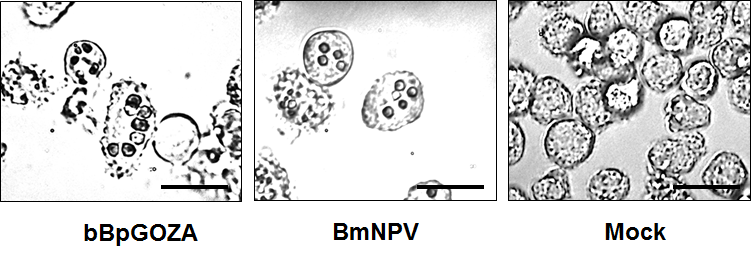

Supplement: S2 Fig — Cells were transfected with bBpGOZA DNA or infected with BmNPV at an MOI of 1 PFU/cell. Bar markers represent 20 ㎛. (TIF) [file pone.0221594.s002.tif]

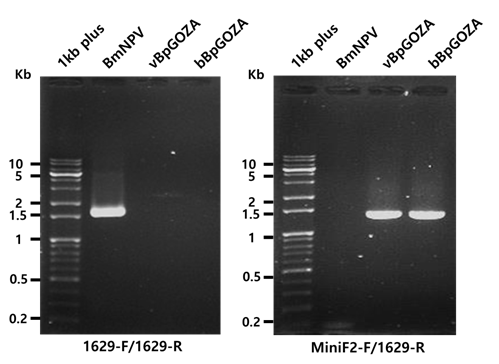

Supplement: S3 Fig — PCR amplifications were performed for BmNPV, vBpGOZA and bBpGOZA DNA using each primer shown Fig 1 and Table 1. (TIF) [file pone.0221594.s003.tif]

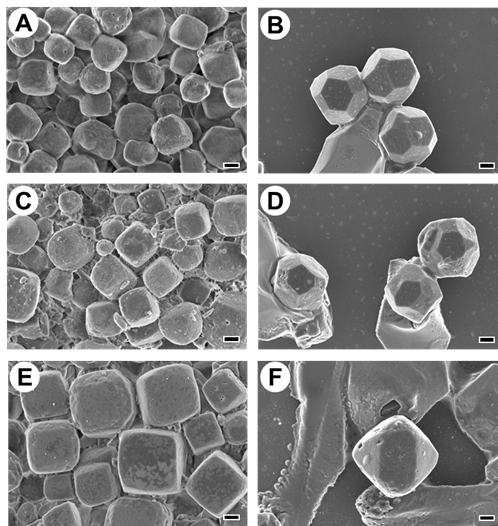

Supplement: S4 Fig — Scanning electron micrographs of purified OBs of AcMNPV (A), BmNPV (B), vApEGFP (C), vBpEGFP (D), vApGOZA (E) and vBpGOZA (F). Bar markers represent 1 μm. (TIF) [file pone.0221594.s004.tif]
