# Supplementary material for: Baculovirus entire ORF1629 is not essential for viral replication
Source: PLoS One. 2019 Aug 22;14(8):e0221594. doi: 10.1371/journal.pone.0221594 (PMC6706055; doi:10.1371/journal.pone.0221594)
Supplement: S5 Fig — ORF603, miniF, kanamycin resistance and defective ORF1629 genes were shown. Nucleotides of 1223 to 1629 from ORF1629 were missed. (DOCX) [file pone.0221594.s005.docx]

**ORF603🡪**

1 AATAATAAAA CAATTATAAA TGCTAAATTT GTTTTTTATT AACGATACAA ACCAAACGCA

61 ACAAGAACAT TTGTAGTATT ATCTATAATT GAAAACGCGT AGTTATAATC GCTGAGGTAA

121 TATTTAAAAT CATTTTCAAA TGATTCACAG TTAATTTGCG ACAATATAAT TTTATTTTCA

181 CATAAACTAG ACGCCTTGTC GTCTTCTTCT TCGTATTCCT TCTCTTTTTC ATTTTTCTCC

241 TCATAAAAAT TAACATAGTT ATTATCGTAT CCATATATGT ATCTATCGTA TAGAGTAAAT

301 TTTTTGTTGT CATAAATATA TATGTCTTTT TTAATGGGGT GTATAGTACC GCTGCGCATA

361 GTTTTTCTGT AATTTACAAC AGTGCTATTT TCTGGTAGTT CTTCGGAGTG TGTTGCTTTA

421 ATTATTAAAT TTATATAATC AATGAATTTG GGATCGTCGG TTTTGTACAA TATGTTGCCG

481 GCATAGTACG CAGCTTCTTC TAGTTCAATT ACACCATTTT TTAGCAGCAC CGGATTAACA

541 TAACTTTCCA AAATGTTGTA CGAACCGTTA AACAAAAACA GTTCACCTCC CTTTTCTATA

601 CTATTGTCTG CGAGCAGTTG TTTGTTGTTA AAAATAACAG CCATTGTAAT GAGACGCACA

661 AACTAATATC ACAAACTGGA AATGTCTATC AATATATAGT TGCTGATATC ATGGAGATAA

**polyhedrin promoter 🡪**

721 TTAAAATGAT AACCATCTCG CAAATAAATA AGTATTTTAC TGTTTTCGTA ACAGTTTTGT

**Kanamycin resistance gene 🡪**

781 AATAAAAAAA CCTATAAATT TAAATGATCG GTACCAGATC TTCTAGATTC GAACTCGAGG

841 CCTGCAGGGG GGGGGGGGCG CTGAGGTCTG CCTCGTGAAG AAGGTGTTGC TGACTCATAC

901 CAGGCCTGAA TCGCCCCATC ATCCAGCCAG AAAGTGAGGG AGCCACGGTT GATGAGAGCT

961 TTGTTGTAGG TGGACCAGTT GGTGATTTTG AACTTTTGCT TTGCCACGGA ACGGTCTGCG

1021 TTGTCGGGAA GATGCGTGAT CTGATCCTTC AACTCAGCAA AAGTTCGATT TATTCAACAA

1081 AGCCGCCGTC CCGTCAAGTC AGCGTAATGC TCTGCCAGTG TTACAACCAA TTAACCAATT

1141 CTGATTAGAA AAACTCATCG AGCATCAAAT GAAACTGCAA TTTATTCATA TCAGGATTAT

1201 CAATACCATA TTTTTGAAAA AGCCGTTTCT GTAATGAAGG AGAAAACTCA CCGAGGCAGT

1261 TCCATAGGAT GGCAAGATCC TGGTATCGGT CTGCGATTCC GACTCGTCCA ACATCAATAC

1321 AACCTATTAA TTTCCCCTCG TCAAAAATAA GGTTATCAAG TGAGAAATCA CCATGAGTGA

1381 CGACTGAATC CGGTGAGAAT GGCAAAAGCT TATGCATTTC TTTCCAGACT TGTTCAACAG

1441 GCCAGCCATT ACGCTCGTCA TCAAAATCAC TCGCATCAAC CAAACCGTTA TTCATTCGTG

1501 ATTGCGCCTG AGCGAGACGA AATACGCGAT CGCTGTTAAA AGGACAATTA CAAACAGGAA

1561 TCGAATGCAA CCGGCGCAGG AACACTGCCA GCGCATCAAC AATATTTTCA CCTGAATCAG

1621 GATATTCTTC TAATACCTGG AATGCTGTTT TCCCGGGGAT CGCAGTGGTG AGTAACCATG

1681 CATCATCAGG AGTACGGATA AAATGCTTGA TGGTCGGAAG AGGCATAAAT TCCGTCAGCC

1741 AGTTTAGTCT GACCATCTCA TCTGTAACAT CATTGGCAAC GCTACCTTTG CCATGTTTCA

1801 GAAACAACTC TGGCGCATCG GGCTTCCCAT ACAATCGATA GATTGTCGCA CCTGATTGCC

1861 CGACATTATC GCGAGCCCAT TTATACCCAT ATAAATCAGC ATCCATGTTG GAATTTAATC

1921 GCGGCCTCGA GCAAGACGTT TCCCGTTGAA TATGGCTCAT AACACCCCTT GTATTACTGT

1981 TTATGTAAGC AGACAGTTTT ATTGTTCATG ATGATATATT TTTATCTTGT GCAATGTAAC

**MiniF 🡪**

2041 ATCAGAGATT TTGAGACACA ACGTGGCTTT CCCCCCCCCC CCTGCAGGAT CAACGTGCCG

2101 GCACGGCCTG GGTAACCAGG TATTTTGTCC ACATAACCGT GCGCAAAATG TTGTGGATAA

2161 GCAGGACACA GCAGCAATCC ACAGCAGGCA TACAACCGCA CACCGAGGTT ACTCCGTTCT

2221 ACAGGTTACG ACGACATGTC AATACTTGCC CTTGACAGGC ATTGATGGAA TCGTAGTCTC

2281 ACGCTGATAG TCTGATCGAC AATACAAGTG GGACCGTGGT CCCAGACCGA TAATCAGACC

2341 GACAACACGA GTGGGATCGT GGTCCCAGAC TAATAATCAG ACCGACGATA CGAGTGGGAC

2401 CGTGGTCCCA GACTAATAAT CAGACCGACG ATACGAGTGG GACCGTGGTT CCAGACTAAT

2461 AATCAGACCG ACGATACGAG TGGGACCGTG GTCCCAGACT AATAATCAGA CCGACGATAC

2521 GAGTGGGACC ATGGTCCCAG ACTAATAATC AGACCGACGA TACGAGTGGG ACCGTGGTCC

2581 CAGTCTGATT ATCAGACCGA CGATACGAGT GGGACCGTGG TCCCAGACTA ATAATCAGAC

2641 CGACGATACG AGTGGGACCG TGGTCCCAGA CTAATAATCA GACCGACGAT ACGAGTGGGA

2701 CCGTGGTCCC AGTCTGATTA TCAGACCGAC GATACAAGTG GAACAGTGGG CCCAGAGAGA

2761 ATATTCAGGC CAGTTATGCT TTCTGGCCTG TAACAAAGGA CATTAAGTAA AGACAGATAA

2821 ACGTAGACTA AAACGTGGTC GCATCAGGGT GCTGGCTTTT CAAGTTCCTT AAGAATGGCC

2881 TCAATTTTCT CTATACACTC AGTTGGAACA CGAGACCTGT CCAGGTTAAG CACCATTTTA

2941 TCGCCCTTAT ACAATACTGT CGCTCCAGGA GCAAACTGAT GTCGTGAGCT TAAACTAGTT

3001 CTTGATGCAG ATGACGTTTT AAGCACAGAA GTTAAAAGAG TGATAACTTC TTCAGCTTCA

3061 AATATCACCC CAGCTTTTTT CTGCTCATGA AGGTTAGATG CCTGCTGCTT AAGTAATTCC

3121 TCTTTATCTG TAAAGGCTTT TTGAAGTGCA TCACCTGACC GGGCAGATAG TTCACCGGGG

3181 TGAGAAAAAA GAGCAACAAC TGATTTAGGC AATTTGGCGG TGTTGATACA GCGGGTAATA

3241 ATCTTACGTG AAATATTTTC CGCATCAGCC AGCGCAGAAA TATTTCCAGC AAATTCATTC

3301 TGCAATCGGC TTGCATAACG CTGACCACGT TCATAAGCAC TTGTTGGGCG ATAATCGTTA

3361 CCCAATCTGG ATAATGCAGC CATCTGCTCA TCATCCAGCT CGCCAACCAG AACACGATAA

3421 TCACTTTCGG TAAGTGCAGC AGCTTTACGA CGGCGACTCC CATCGGCAAT TTCTATGACA

3481 CCAGATACTC TTCGACCGAA CGCCGGTGTC TGTTGACCAG TCAGTAGAAA AGAAGGGATG

3541 AGATCATCCA GTGCGTCCTC AGTAAGCAGC TCCTGGTCAC GTTCATTACC TGACCATACC

3601 CGAGAGGTCT TCTCAACACT ATCACCCCGG AGCACTTCAA GAGTAAACTT CACATCCCGA

3661 CCACATACAG GCAAAGTAAT GGCATTACCG CGAGCCATTA CTCCTACGCG CGCAATTAAC

3721 GAATCCACCA TCGGGGCAGC TGGTGTCGAT AACGAAGTAT CTTCAACCGG TTGAGTATTG

3781 AGCGTATGTT TTGGAATAAC AGGCGCACGC TTCATTATCT AATCTCCCAG CGTGGTTTAA

3841 TCAGACGATC GAAAATTTCA TTGCAGACAG GTTCCCAAAT AGAAAGAGCA TTTCTCCAGG

3901 CACCAGTTGA AGAGCGTTGA TCAATGGCCT GTTCAAAAAC AGTTCTCATC CGGATCTGAC

3961 CTTTACCAAC TTCATCCGTT TCACGTACAA CATTTTTTAG AACCATGCTT CCCCAGGCAT

4021 CCCGAATTTG CTCCTCCATC CACGGGGACT GAGAGCCATT ACTATTGCTG TATTTGGTAA

4081 GCAAAATACG TACATCAGGC TCGAACCCTT TAAGATCAAC GTTCTTGAGC AGATCACGAA

4141 GCATATCGAA AAACTGCAGT GCGGAGGTGT AGTCAAACAA CTCAGCAGGC GTGGGAACAA

4201 TCAGCACATC AGCAGCACAT ACGACATTAA TCGTGCCGAT ACCCAGGTTA GGCGCGCTGT

4261 CAATAACTAT GACATCATAG TCATGAGCAA CAGTTTCAAT GGCCAGTCGG AGCATCAGGT

4321 GTGGATCGGT GGGCAGTTTA CCTTCATCAA ATTTGCCCAT TAACTCAGTT TCAATACGGT

4381 GCAGAGCCAG ACAGGAAGGA ATAATGTCAA GCCCCGGCCA GCAAGTGGGC TTTATTGCAT

4441 AAGTGACATC GTCCTTTTCC CCAAGATAGA AAGGCAGGAG AGTGTCTTCT GCATGAATAT

4501 GAAGATCTGG TACCCATCCG TGATACATTG AGGCTGTTCC CTGGGGGTCG TTACCTTCCA

4561 CGAGCAAAAC ACGTAGCCCC TTCAGAGCCA GATCCTGAGC AAGATGAACA GAAACTGAGG

4621 TTTTGTAAAC GCCACCTTTA TGGGCAGCAA CCCCGATCAC CGGTGGAAAT ACGTCTTCAG

4681 CACGTCGCAA TCGCGTACCA AACACATCAC GCATATGATT AATTTGTTCA ATTGTATAAC

4741 CAACACGTTG CTCAACCCGT CCTCGAATTT CCATATCCGG GTGCGGTAGT CGCCCTGCTT

4801 TCTCGGCATC TCTGATAGCC TGAGAAGAAA CCCCAACTAA ATCCGCTGCT TCACCTATTC

4861 TCCAGCGCCG GGTTATTTTC CTCGCTTCCG GGCTGTCATC ATTAAACTGT GCAATGGCGA

4921 TAGCCTTCGT CATTTCATGA CCAGCGTTTA TGCACTGGTT AAGTGTTTCC ATGAGTTTCA

4981 TTCTGAACAT CCTTTAATCA TTGCTTTGCG TTTTTTTATT AAATCTTGCA ATTTACTGCA

5041 AAGCAACAAC AAAATCGCAA AGTCATCAAA AAACCGCAAA GTTGTTTAAA ATAAGAGCAA

5101 CACTACAAAA GGAGATAAGA AGAGCACATA CCTCAGTCAC TTATTATCAC TAGCGCTCGC

5161 CGCAGCCGTG TAACCGAGCA TAGCGAGCGA ACTGGCGAGG AAGCAAAGAA GAACTGTTCT

5221 GTCAGATAGC TCTTACGCTC AGCGCAAGAA GAAATATCCA CCGTGGGAAA AACTCCAGGT

5281 AGAGGTACAC ACGCGGATAG CCAATTCAGA GTAATAAACT GTGATAATCA ACCCTCATCA

5341 ATGATGACGA ACTAACCCCC GATATCAGGT CACATGACGA AGGGAAAGAG AAGGAAATCA

5401 ACTGTGACAA ACTGCCCTCA AATTTGGCTT CCTTAAAAAT TACAGTTCAA AAAGTATGAG

5461 AAAATCCATG CAGGCTGAAG GAAACAGCAA AACTGTGACA AATTACCCTC AGTAGGTCAG

5521 AACAAATGTG ACGAACCACC CTCAAATCTG TGACAGATAA CCCTCAGACT ATCCTGTCGT

5581 CATGGAAGTG ATATCGCGGA AGGAAAATAC GATATGAGTC GTCTGGCGGC CTTTCTTTTT

5641 CTCAATGTAT GAGAGGCGCA TTGGAGTTCT GCTGTTGATC TCATTAACAC AGACCTGCAG

5701 GAAGCGGCGG CGGAAGTCAG GCATACGCTG GTAACTTTGA GGCAGCTGGT AACGCTCTAT

5761 GATCCAGTCG ATTTTCAGAG AGACGATGCC TGAGCCATCC GGCTTACGAT ACTGACACAG

5821 GGATTCGTAT AAACGCATGG CATACGGATT GGTGATTTCT TTTGTTTCAC TAAGCCGAAA

5881 CTGCGTAAAC CGGTTCTGTA ACCCGATAAA GAAGGGAATG AGATATGGGT TGATATGTAC

5941 ACTGTAAAGC CCTCTGGATG GACTGTGCGC ACGTTTGATA AACCAAGGAA AAGATTCATA

6001 GCCTTTTTCA TCGCCGGCAT CCTCTTCAGG GCGATAAAAA ACCACTTCCT TCCCCGCGAA

6061 ACTCTTCAAT GCCTGCCGTA TATCCTTACT GGCTTCCGCA GAGGTCAATC CGAATATTTC

6121 AGCATATTTA GCAACATGGA TCTCGCAGAT ACCGTCATGT TCCTGTAGGG TGCCATCAGA

6181 TTTTCTGATC TGGTCAACGA ACAGATACAG CATACGTTTT TGATCCCGGG AGAGACTATA

6241 TGCCGCCTCA GTGAGGTCGT TTGACTGGAC GATTCGCGGG CTATTTTTAC GTTTCTTGTG

6301 ATTGATAACC GCTGTTTCCG CCATGACAGA TCCATGTGAA GTGTGACAAG TTTTTAGATT

6361 GTCACACTAA ATAAAAAAGA GTCAATAAGC AGGGATAACT TTGTGAAAAA ACAGCTTCTT

6421 CTGAGGGCAA TTTGTCACAG GGTTAAGGGC AATTTGTCAC AGACAGGACT GTCATTTGAG

6481 GGTGATTTGT CACACTGAAA GGGCAATTTG TCACAACACC TTCTCTAGAA CCAGCATGGA

6541 TAAAGGCCTA CAAGGCGCTC TAAAAAAGAA GATCTAAAAA CTATAAAAAA AATAATTATA

6601 AAAATATCCC CGTGGATAAG TGGATAACCC CAAGGGAAGT TTTTTCAGGC ATCGTGTGTA

6661 AGCAGAATAT ATAAGTGCTG TTCCCTGGTG CTTCCTCGCT CACTCGAGGG CTTCGCCGTC

6721 GCTCGACTGC GGCGAGCCTA CTGGCTGTAA AAGGACAGAC CACATCATGG TTCTGTGTTC

6781 ATTAGGTTGT TCTGTCCATT GCTGACATAA TCCGCTCCAC TTCAACGTAA CACCGCACGA

6841 AGATTTCTAT TGTTCCTGAA GGCATATTCA AATCGTTTTC GTTACCGCTT GCAGGCATCA

6901 TGACAGAACA CTACTTCCTA TAAACGCTAC ACAGGCTCCT GAGATTAATA ATGCGGATCT

6961 CTACGATAAT GGGAGATTTT CCCGACTGTT TCGTTCGCTT CTCAGTGGAT AACAGCCAGC

7021 TTCTCTGTTT AACAGACAAA AACAGCATAT CCACTCAGTT CCACATTTCC ATATAAAGGC

7081 CAAGGCATTT ATTCTCAGGA TAATTGTTTC AGCATCGCAA CCGCATCAGA CTCCGGCATC

7141 GCAAACTGCA CCCGGTGCCG GGCAGCCACA TCCAGCGCAA AAACCTTCGT GTAGACTTCC

7201 GTTGAACTGA TGGACTTATG TCCCATCAGG CTTTGCAGAA CTTTCAGCGG TATACCGGCA

7261 TACAGCATGT GCATCGCATA GGAATGGCGG AACGTATGTG GTGTGACCGG AACAGAGAAC

7321 GTCACACCGT CAGCAGCAGC GGCGGCAACC GCCTCCCCAA TCCAGGTCCT GACCGTTCTG

7381 TCCGTCACTT CCCAGATCCG CGCTTTCTCT GTCCTTCCTG TGCGACGGTT ACGCCGCTCC

7441 ATGGGTATTT TCAGTTGTGC CACCATCGTC TGCAGCTGGC TGACGTACCA GGAGTCAGAG

7501 AGCGGAACCA GCCGGTGAGT CTGCTGACCG GCGGGCATTC TCCCCGCCGT CCTGGCAGCT

7561 TTTTCGGTCC GTTGTTTCAG GGTCGCAAGC TGCACAAACG GATACGGAGG CGCAAGCGAA

7621 AAATCCCCCC GCGTCAGCGC CAGTGCTTCA TTAATGCGTG CTCCGGTGTT CCACAGTGTG

7681 GCCAGCAGCA TCTTGCGGTG CAGATCCGGG ACGTAATGGA GCAGGGCACT CACTTCCGGA

7741 GCCAGCAGAT ATTTTGGCAG TTCATCATGG ACCATCGACA TCTGGCGAAG TGCCAGAGCT

7801 GCCGGATAAT CAATGGCAAC AGGCAGCGAT GCAGGCTGCC CGGCAGAATA CACTGCCGGT

7861 ACCATGACTG CAGACTGGCT GTGTATAACG GAGCCTGACA TTTATATTCC CCAGAACATC

7921 AGGTTAATGG CGTTTTTGAT GTCATTTTCG CGGTGGCTGA GATCAGCCAC TTCTTCCCCG

7981 ATAACGGAGA CCGGCACACT GGCCATATCG GTGGTCATCA TGCGCCAGCT TTCATCCCCG

8041 ATATGCACCA CCGGGTAAAG TTCACGGGAG ACTTTATCTG ACAGCAGACG TGCACTGGCC

8101 AGGGGGATCA CCATCCGTCG CCCGGGCGTG TCAATAATAT CACTCTGTAC ATCCACAAAC

8161 AGACGATAAC GGCTCTCTCT TTTATAGGTG TAAACCTTAA ACTGCATTTC ACCAGTCCCT

8221 GTTCTCGTCA GCAAAAGAGC CGTTCATTTC AATAAACCGG GCGACCTCAG CCATCCCTTC

8281 CTGATTTTCC GCTTTCCAGC GTTCGGCACG CAGACGACGG GCTTCATTCT GCATGGTTGT

8341 GCTTACCAGA CCGGAGATAT TGACATCATA TGCCTTGAGC AACTGATAGC TGTCGCTGTC

8401 AACTGTCACT GTAATACGCT GCTTCATAGC ACACCTCTTT TTGACATACT TCGGGTATAC

8461 ATATCAGTAT ATATTCTTAT ACCGCAAAAA TCAGCGCGCA AATACGCATA CTGTTATCTG

8521 GCTTTTAGTA AGCCTTATGT ATTTTACCTT TCGTTATGTT AATTCGAATT CGAGCTCCAC

**defective ORF1629 🡪**

8581 CGCGGTGGCG GCCGCTCTAG AACTAGTGGA TCCCTGCAGG CCTCGAAGCT TGGACATATT

8641 TAACATCGGG CGTGTTAGCT TTATTAGGCC GATTATCGTC GTCGTCCCAA CCCTCGTCGT

8701 TAGAAGTTGC TTCCGAAGAC GATTTTGCCA TAGCCACACG ACGCCTATTA ATTGTGTCGG

8761 CTAACACGTC CGCGATCAAA TTTGTAGTTG AGCTTTTTGG AATTATTTCT GATTGCGGGC

8821 GTTTTTGGGC GGGTTTCAAT CTAACTGTGC CCGATTTTAA TTCAGACAAC ACGTTAGAAA

8881 GCGATGGTGC AGGCGGTGGT AACATTTCAG ACGGCAAATC TACTAATGGC GGCGGTGGTG

8941 GAGCTGATGA TAAATCTACC ATCGGTGGAG GCGCAGGCGG GGCTGGCGGC GGAGGCGGAG

9001 GCGGAGGTGG TGGCGGTGAT GCAGACGGCG GTTTAGGCTC AAATGTCTCT TTAGGCAACA

9061 CAGTCGGCAC CTCAACTATT GTACTGGTTT CGGGCGCCGT TTTTGGTTTG ACCGGTCTGA

9121 GACGAGTGCG ATTTTTTTCG TTTCTAATAG CTTCCAACAA TTGTTGTCTG TCGTCTAAAG

9181 GTGCAGCGGG TTGAGGTTCC GTCGGCATTG GTGGAGCGGG CGGCAATTCA GACATCGATG

9241 GTGGTGGTGG TGGTGGAGGC GCTGGAATGT TAGGCACGGG AGAAGGTGGT GGCGGCGGTG

9301 CCGCCGGTAT AATTTGTTCT GGTTTAGTTT GTTCGCGCAC GATTGTGGGC ACCGGCGCAG

9361 GCGCCGCTGG CTGCACAACG GAAGGTCGTC TGCTTCGAGG CAGCGCTTGG GGTGGTGGCA

9421 ATTCAATATT ATAATTGGAA TACAAATCGT AAAAATCTGC TATAAGCATT GTAATTTCGC

9481 TATCGTTTAC CGTGCCGATA TTTAACAACC GCTCAATGTA AGCAATTGTA TTGTAAAGAG

9541 ATTGTCTCAA GCTCGGATCC CGCACGCCGA TAACAAGCCT TTTCATTTTT ACTACAGCAT

9601 TGTAGTGGCG AGACACTTCG CTGTCGTCGA CGTACATGTA TGCTTTGTTG TCAAAAACGT

9661 CGTTGGCAAG CTTTAAAATA TTTAAAAGAA CATCTCTGTT CAGCACCACT GTGTTGTCGT

9721 AAATGTTGTT TTTGATAATT TGCGCTTCCG CAGTATCGAC ACGTTCAAAA AATTGATGCG

9781 CATCAATTTT GTTGTTCCTA TTATTGAATA AATAAGATTG TACAGATTCA TATCTACGAT

9841 TCGTCATGGC CACCACAAAT GCTACGCTGC AAACGCTGGT ACAATTTTAC GAAAACTGCA

9901 AAAACGTCA
